# Temperament Behaviours in Individually Tested Sheep Are Not Related to Behaviours Expressed in the Presence of Conspecifics

**DOI:** 10.3390/ani14010155

**Published:** 2024-01-02

**Authors:** Leigh Atkinson, Rebecca E. Doyle, Ellen C. Jongman

**Affiliations:** 1 The Animal Welfare Science Centre, Faculty of Science, The University of Melbourne, Parkville, VIC 3052, Australia; 2 Jeanne Marchig International Centre for Animal Welfare Education, The Royal (Dick) School of Veterinary Studies, University of Edinburgh, Edinburgh EH9 1RS, UK

**Keywords:** vocalisations, locomotion, vigilance, social isolation

## Abstract

**Simple Summary:**

To identify sheep temperament, researchers often test sheep individually. Under these conditions, vocalisations and locomotory behaviours in sheep are highly repeatable over time and are therefore commonly used as behavioural measures of temperament. However, sheep are highly social animals that are usually managed in flocks where behaviour may be influenced by the presence of other sheep. We tested 220 Merino lambs individually and in small groups of four to identify if the behaviour expressed in the two different social situations was related. We found that vocalisations and locomotory behaviours were not related between the two social situations; however, vigilance behaviours were. Vocalisations were rarely performed when other sheep were present, suggesting that this behaviour is a response to being alone. Vigilance may be more suitable to classify the temperament of sheep that will be managed in flocks.

**Abstract:**

Individual behavioural testing in sheep is common; however, outcomes may be misleading as they are a highly gregarious species that is usually managed in groups. We investigated whether behaviour expressed by 3–4-month-old Merino lambs (*n* = 220) in social isolation was related to their behaviour towards the same stimuli when three other conspecifics were present, and if measures of temperament (vocalisations and locomotory behaviours) were repeatable across both social situations. Expression of all behaviours were reduced when conspecifics were present, and vocalisations were rarely performed in social groups, suggesting that this behaviour is a response to social isolation. Similarities across the two social situations, in ranked order of how individual lambs expressed each behaviour, indicate that vigilance and attentional orienting towards a human were repeatable (*p* < 0.001), as was vigilance in a startle test (*p* < 0.05). However, no clear relationship between behaviours expressed across the two social situations was found. The results of this study suggest that testing sheep individually should be conducted with caution where the outcome is applied to animals managed in groups. Vigilance shows promise as a measure of an underlying trait that is stable across social contexts.

## 1. Introduction

Temperament in farm animals is defined as an individual’s “inherent potential to respond in a particular way to a stressful stimulus” [1]. A key criterion for identifying that a behaviour is indicative of temperament is that the performance of the behaviour remains stable (or repeatable) over time and/or across situations [2]. Sih et al. [3] describes a situation as “a given set of conditions at one point in time”, such as the level of predation risk, food availability, life stage, or differing social conditions. The stability of a behaviour does not necessarily refer to maintaining the same degree of expression, but rather that the degree of expression relative to the conspecifics remains similar [2]. For example, while testing sheep in groups is known to reduce the overall expression of behaviours compared to individual testing, if individuals within the same cohort are ranked similarly across both situations, the performance of that behaviour could be considered stable. The temporal stability, or repeatability, of vocalisations and locomotory behaviours across different tests in sheep is well documented [4,5,6,7,8,9] and these behaviours are therefore commonly used as measures of temperament in sheep. However, their stability across situations requires further exploration. In a previous study, we confirmed the repeatability of vocalisations over time under different testing conditions involving three response-eliciting stimuli [10]. This indicated the presence of a domain-general temperament trait which we labelled sociability, defined as the response to either the presence or absence of conspecifics [2]. We hypothesised that vocalisations had been expressed in response to the absence of conspecifics, as all tests were conducted under social isolation. We were interested to learn if this behaviour would be performed similarly when conspecifics were present and if it was therefore repeatable across different social situations.

Temperament is strongly expressed under novel, risky, or challenging conditions [2]. As sheep are a highly gregarious species, a key characteristic that has enabled their domestication, social isolation is very challenging. It elicits both behavioural and physiological changes indicative of stress [11,12] and is more stressful than the sudden appearance of a stimulus when conspecifics are present [13]. When conspecifics are present, behavioural responses towards stimuli are significantly reduced, which leads to low behavioural variability within a group [13,14]. As a certain degree of variability is necessary to differentiate temperament types, social isolation is a more suitable condition for temperament testing [14]. However, in their review, Forkman et al. [15] reiterate that for farmed animals, social isolation likely plays a larger role in the reactions observed during behavioural tests than the environment or stimuli of interest. Therefore, conclusions drawn from strong reactions under social isolation may not be applicable for a species, such as sheep that are normally managed, and would normally encounter challenging conditions when in a group [15]. And while behavioural indicators of temperament in sheep tested in social isolation have been linked with differences in growth, maternal ability, and milk production (see [1] for a review), the mechanism of these relationships remains unclear. 

The aim of this study was to explore the relationship between temperament and fear-related behaviours towards different stimuli in sheep, across two different social situations: in the absence and presence of conspecifics. Our first objective was to identify if individual behaviours were repeatable across the two situations by comparing the ranked order of each lamb, to see if individuals were ranked similarly in both situations. While we expected that all behaviours would be expressed less when conspecifics were present, we hypothesised that measures of temperament (locomotion in the presence of a human and vocalisations) would show similar rankings across social situations. Our second objective was to identify if each behaviour expressed in social isolation was related to any other behaviour expressed when conspecifics were present. We wanted to better understand if the way sheep behave when socially isolated is indicative of the way they behave in a group setting. To do this, we again compared the ranked order of behavioural expression across the two situations, and we also compared behavioural profiles that were generated for each social situation. We had no a priori assumptions for this objective. As sexual dimorphism is known to influence behavioural responses in sheep [16,17,18], the effect of sex was also considered. This study could inform whether individual testing is suitable for a highly gregarious species that is usually managed in groups.

## 2. Materials and Methods

### 2.1. Ethics Statement

This study was approved by the University of Melbourne’s Faculty of Veterinary and Agricultural Sciences Animal Ethics Committee, ethical review number 1914990.2.

### 2.2. Animals and Housing

This study was conducted over 16 days in November 2019 at the University of Melbourne’s research facility at Dookie, Victoria, Australia. The average daily maximum temperature for this region was 28.8 °C over the study period, with a total of 4.6 mm of rain.

Two-hundred and forty Merino lambs (120 ewe-lambs, 120 castrate males) were weaned at 1–2 months of age and underwent behavioural testing two months later when 3–4 months old (19–39 kg). The 240 study lambs were randomly selected from a larger cohort by alternately selecting individuals from the front, central, and rear areas of a holding yard. A random number generator was used to allocate all lambs into three home groups: all females (*n* = 80), all males (*n* = 80), and one with equal numbers of each sex (*n* = 80). Each home group was housed in its own large outdoor pen, measuring approximately 2000 m^2^, where lambs had ad libitum access to water and lucerne hay for the duration of the trial. Lambs were provided with two days to become familiar with both their environment and their pen mates and testing commenced on the third day after their arrival.

### 2.3. Behavioural Testing

**Figure 1:**
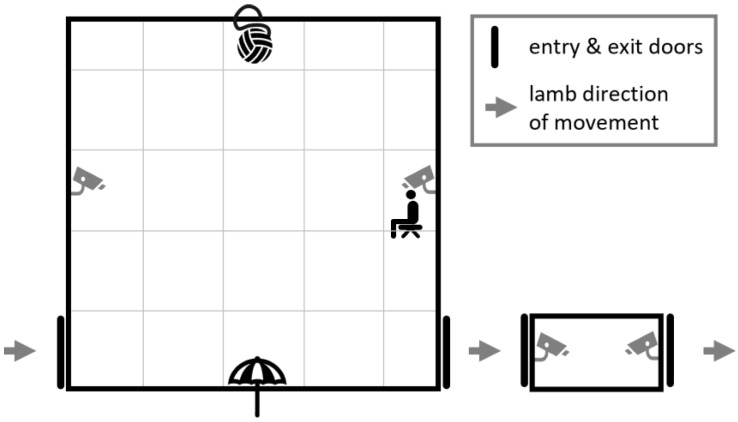
Layout of the arena and isolation box indicating their positions respective to each other; direction of lamb movement through the tests; positions of the ball and umbrella startle objects; where the human sat; and positions of the doors and cameras.

This study used the same three behavioural tests and measures of temperament and reactivity as Atkinson et al. [10] and repeated these tests under two different social conditions: in the absence and presence of conspecifics. Each lamb was tested once individually (IND) and once with three other conspecifics (GRP) which occurred over two consecutive days. A group size of four was chosen as it was small enough to maximise the total number of groups, while still eliciting significant differences in behaviour when compared with individually tested sheep [14]. It also allowed for sex to be balanced in the mixed sex groups. The order of testing for each social condition was alternated for each home group to account for possible habituation to the tests by the second day. Testing was conducted in a 5 m × 5 m × 2 m plywood arena (human and startle/novel object tests; Figure 1) and a separate 1.25 m × 0.5 m × 1 m small, solid-sided weigh crate (isolation box). Two GoPro HERO3 Silver edition cameras (GoPro, Inc., San Mateo, CA, USA) were attached to the tops of opposing walls of the arena and two more at floor level of the isolation box to record all behaviours, which were later logged from video playback, except for vocalisations, which were tallied on testing days. On the evening prior to the first day of testing, 40 lambs from one group were calmly walked from their pen for approximately 150 m to a holding area surrounding the main testing area. Lambs were rested here overnight with ad libitum access to water and hay. Lambs also had access to move freely through a raceway and the testing arena; however, no lambs from any group entered these areas voluntarily. Lambs remained in the holding area overnight between testing days 1 and 2. Once both testing days were complete, all 40 lambs were walked calmly back to their home pen and the next 40 lambs were walked up. This was repeated over 13 days until all 240 lambs had been tested (testing was paused for one day during the trial between the end of one group and the start of the next due to an extreme weather event).

On IND testing days, lambs were drafted one at a time and ear identification tags were scanned and recorded immediately prior to being ushered into the arena. The first test began once the lamb had been in the arena for thirty seconds. IND testing consisted of three stimulus tests: exposure to a stationary human, exposure to a startling/novel object, and confinement within an isolation box. The behaviours recorded for each test are listed in Table 1. On GRP testing days, four lambs at a time were drafted into a race adjacent to the test arena, with groups from the larger mixed sex group always consisting of two males and two females. Each lamb was spray marked with a small, coloured dot on its head for individual identification on video and ear tag identification numbers were recorded against the colour. All four lambs were ushered into the arena together and the first test began after thirty seconds. GRP testing consisted of the human and startle tests only (see Table 1 for behaviours measured).

#### 2.3.1. Stationary Human (H) Test

This test was used to measure human-directed fear responses and was based on a modified version of a forced approach test [10,19] with an additional modification. As a moving human could prompt highly reactive lambs to sprint across the arena and jump against the walls, this study used a stationary human to ensure the safety of both the lambs and the human. After the lamb/s had been in the arena for 30 s, an unfamiliar human quietly entered, moved slowly 2.5 m to the centre of one wall, and sat on a small stool they had carried in with them. Timing began once the human was seated and continued for 4 min. The human looked at a position on the ground approximately 1 m in front of them and did not move, even if approached or nudged by a lamb. After 4 min, the human quietly stood and slowly exited the arena with the stool.

#### 2.3.2. Startle (S)/Novel Object Test

This test measures both the initial response to being startled and subsequent response to the novel startle object and was included to measure non-human directed behavioural reactivity. Once the human had exited and closed the arena door, an object was deployed from one of the walls perpendicular to the wall the human had sat against in order to startle the lambs. To ensure lambs did not anticipate the startle the second time they were tested, two different startle stimuli were used and were deployed from opposing walls. On the first day, an umbrella was poked through a previously covered hole in the middle of one wall. For IND testing, once the lamb had oriented its head toward the umbrella, it was popped open in a swift motion and left to rest, open, against the wall. In the GRP condition, as not all lambs directed their attention to the unopened umbrella when it first appeared, a decision was made that the umbrella would be opened when at least two lambs had oriented their heads toward it. On the second day, the startle object was a ball tethered to the top of the opposing wall with a bungee cord. The ball was first held at the top of the wall by someone standing on the outside of the arena until again, either the individual or at least two of the GRP lambs oriented their heads towards it. It was then tossed into the arena and bounced against the wall a few times until it came to rest. Despite some lambs not orienting their heads towards the test stimuli, both items were clearly audible as they were deployed, and it is reasonable to assume all lambs were aware of both at the time of deployment. The test continued for 4 min to observe the lamb’s behaviours in response to the novel objects. At the completion of the startle test, IND lambs were ushered into the isolation box, and GRP lambs were ushered out and allowed to move freely to the holding area.

#### 2.3.3. Isolation Box (IB)/Temperament Test

The Isolation Box test is commonly used to categorise temperament in sheep and was used in the current study as a reference test and only for the IND condition, due to the size of the box and the nature of the test. On completion of the startle test, individual lambs were ushered from the arena into the isolation box located 1 m away. Once inside, the rear door to the box was closed and recording was conducted for 2 min. At the completion of this test, lambs were released from the front of the box and allowed to make their way freely to the holding area.

### 2.4. Statistical Analysis

All data were analysed using R Statistical Software (v4.1.3; R Core Team [20]) and *p*-values < 0.05 were considered significant. 

#### 2.4.1. Determining the Repeatability of Behaviours across Social Conditions

To determine the stability of behaviours across social conditions, the 13 behaviours that were measured in both conditions for the human and startle tests only were compared. Of the 240 lambs tested, 220 individuals (112 females, 108 males) had a full dataset for this analysis. Behaviours with non-ordinal measures were first ranked, and tied observations were assigned their average rank. For example, if three individuals ranked 20–22 had the same observations, they were all ranked as 21. Ranked data, as opposed to raw data, was compared as it wasn’t the focus of this study to compare changes in the magnitude of responses across the social conditions. Although proximity to stimuli measures were recorded as ordinal responses, the 1 metre differences between levels approximated a linear scale, and so these were analysed with the ranked, non-ordinal measures using linear mixed effects models and the lme4 package [21]. A generalised linear mixed effects model (GLMM) with a cumulative logit link using the ordinal package [22] was used to analyse the remaining ordinal behaviour, startle response. For all models, GRP measurements were fit as outcome variables; IND measurements as baseline covariates; and sex, test day order, and weight at weaning as fixed effects, and each group of four was allocated its own identification number which was included as a random effect. Backwards stepwise selection, eliminating the least statistically significant variable at each step, was applied to the fixed effects and resulted in sex and weight at weaning being removed from all models and the test day order being removed from locomotion for both arena tests and from proximity to the human.

#### 2.4.2. Exploring the Relationship between Behaviours across Social Situations

Two approaches were used to explore the relationship between behaviours expressed across social conditions. A total of 218 individuals (112 females, 106 males) had a full dataset from all three tests for these analyses. The first approach was to again compare individual rankings across the two social situations, this time between different behaviours. Each GRP behaviour was fit as an outcome variable and each model included all 18 IND behaviours as predictor variables, with sex, weight at weaning, and test day order as fixed effects and group ID as a random effect. Backwards stepwise elimination was again used to determine which predictor variables and fixed effects parameters should be removed from each model. A total of nine models were analysed as vocalisations and escape attempts from the GRP testing were excluded due to a low number of occurrences. As before, non-ordinal measures were ranked and tied values were averaged. The startle response was analysed using a GLMM with cumulative logit link, and all other behaviours were analysed using linear mixed effects models. For the second approach, we compared the behavioural profiles of each individual lamb for each social condition for similarities. To do this, we used the same approach as Atkinson et al. [10] to first identify the underlying temperament traits for each social condition using PCA. A subset of the data containing the 19 IND behaviours and a second subset containing the 13 GRP behaviours were each centred and scaled and the correlation matrix of each subset was assessed. Behaviours with an absolute correlation coefficient <0.3 with all other behaviours were removed from the matrices, which were then used to generate the two PCAs using the stats package [20]. Visual inspection of scree plots was used to determine the number of principal components to be retained and a varimax rotation was performed on each PCA using the psych package [23]. Behaviours within each PC with an absolute loading >0.45 were used for interpretation [24]. Cluster analysis was then used to determine the behavioural profiles of individual lambs. A hierarchical cluster analysis using an Euclidean distance and Ward’s method was performed on the same two centred and scaled data subsets using the cluster package [25]. The NbClust package was used to identify an optimal number of clusters (see Charrad et al. [26] for details of the 30 validation indices used), which was identified as four for the IND data and three for the GRP data. To interpret each cluster and define the behavioural profiles, a k-means cluster analysis using the stats package [20] was performed on each data subset to extract the cluster mean of each behaviour. The temperament traits defined from the PCAs were used to aid the interpretation of the behavioural profiles.

## 3. Results

Descriptive statistics of those behaviours measured across both testing conditions show that for all behaviours, there was a reduction in either the behavioural mean and/or the number of lambs to perform the behaviour in the GRP condition (Table 2). There were 11 lambs in total that vocalised in at least one test in the GRP condition (five females and six males). Of these, only four vocalised in both GRP tests, and of these, only three vocalised across all tests and conditions, ranging between 8 and 72 vocalisations in total.

### 3.1. Repeatability of Behaviours across Social Conditions

Due to the low occurrence of both vocalisations and escape attempts under the GRP condition, the rank order of these behaviours could not be analysed. Of the remaining nine behaviours, four showed evidence of rank stability across the two social conditions (Table 3): Vigilance in both the human (*p* < 0.001) and startle tests (*p* = 0.018); attention towards the stimulus in the human test (*p* < 0.001); and locomotory behaviour in the startle test, measured as the number of steps (*p* = 0.048). The test day order had a significant effect on all behaviours in the startle test except locomotion, as well as on vigilance and attention in the human test. Behavioural responses in the GRP condition were greater for lambs who were tested in groups first, compared with lambs who were tested individually first.

### 3.2. Exploring the Relationship between Behaviours across Social Situations by Comparing Rank Order

Similarities in rank order between the expression of different IND and GRP behaviours were also present (Table 4). A total of nine IND behaviours, including four that have previously shown temporal stability, had a similar rank order to eight GRP behaviours, making a total of ten similar ranking pairs. Of these, one is between behaviours within the startle test, two are between behaviours within the human test, and seven are between behaviours across different tests. The test day order had a significant effect in all but two predictive models (*p* < 0.001), where lambs that were tested in groups first had greater behavioural responses in the GRP condition than lambs that were tested individually first. Lambs in the GRP condition were also more likely to approach the startle stimulus when group testing preceded individual testing, which corresponded with the presentation of the umbrella in the GRP condition. Sex and weight at weaning had a significant effect on attention towards the startle stimuli in the GRP condition, with males being less attentive (−19.8, *p* = 0.03) and heavier lambs more attentive (2.3, *p* = 0.02).

### 3.3. Exploring the Relationship between Behaviours across Social Situations by Comparing Behavioural Profiles

**Figure 2:**
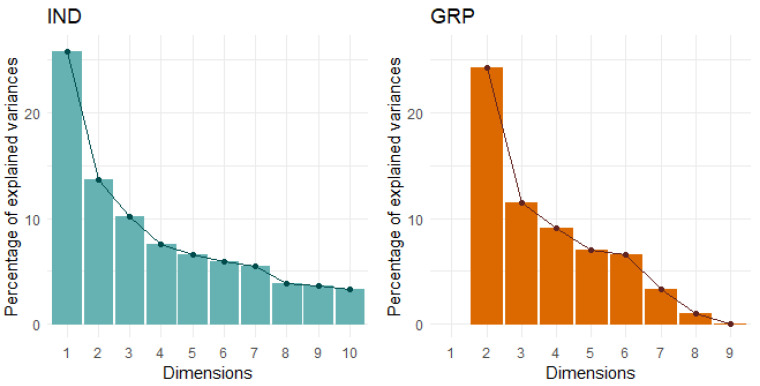
Scree plots for each social condition indicate four principal components should be retained for the IND condition (total 18 behaviours) and three for the GRP condition (total nine behaviours).

The only behaviour in the IND condition that did not have an absolute correlation coefficient >0.3 with any other behaviour was the startle response and so it was excluded from the multivariate analyses. From the PCA of the remaining 18 behaviours, four PCs that described 56% of the total variance were retained (Table 5, Figure 2). As the first three PCs closely matched those identified previously [10], the same labels, which were based on temperament trait categories proposed by Réale et al. [2], were retained. PC1 explained 22% of the total variance and was composed of seven behaviours: Vocalisations from all three tests, interaction behaviours from the human and startle tests, and locomotion from the startle test, which loaded positively, as well as proximity behaviours from the human and startle tests, which loaded negatively. This PC was labelled Sociability/Explore–Avoid. PC2 explained 12% of the total variance and was composed of steps and turns from the isolation box test and the interaction behaviour from the startle test, which all loaded positively. This PC was labelled General Activity. PC3 also explained 12% of the total variance and was composed of the escape and locomotion behaviours from both arena tests, which loaded positively, and attention from both arena tests, which loaded negatively. This PC was labelled Bold–Shy. Lastly, PC4 explained 11% of the variance and comprised the vigilance behaviours from both arena tests, which both loaded positively. This PC was labelled Vigilance.

**Figure 3:**
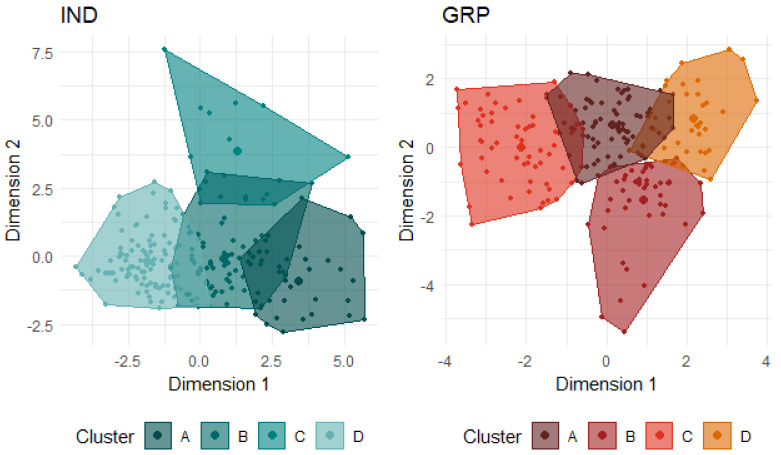
Clustering of the IND data and GRP data each indicate four groups of lambs based on similarities and differences in the expression of behaviours in the respective social conditions. Dimension 1 approximates the first principal component (PC) from each respective dataset and dimension 2 approximates the second PC.

Four clusters were identified as optimal for the IND condition (Table 6, Figure 3). Cluster labels were based on the defining/differentiating behaviours of each group. Cluster A (*n* = 32) consisted of lambs that were highly vocal, came within close proximity to and interacted with both the human and the startle objects, and were moderately active in all tests. This group was labelled the Exploratory group. Lambs in cluster D (*n* = 99) displayed largely opposite expressions of each behaviour. They vocalised the least and stayed the furthest away, but they directed the most attention towards both the human and the startle objects, were the least likely to interact with the stimuli, and were the least active across all three tests. Despite these differences, this group spent a similar length of time vigilant as the lambs from group A. Group D was labelled the Freeze group. Cluster C (*n* = 14) was the most active group across all three tests, particularly in the IB test, and was the group that attempted escape the most. While they were also the most vigilant of the groups, low attention was directed towards both the human and the startle objects. This group was labelled the Active group. Lastly, cluster B (*n* = 73) was largely intermediate of all groups in all behaviours, with the exception of being the least vigilant in both arena tests. This group was labelled as the Head-Down group.

Due to the low occurrences of vocalisations and escape attempts in the GRP condition, these behaviours were also removed from all multivariate analyses and nine behaviours were analysed. Three PCs describing 56% of the total variance were retained (Figure 2, Table 7). The PCs explained 25%, 19%, and 15% of the variance and were comprised of four, three, and two behaviours, respectively. Attention and vigilance behaviours from both arena tests loaded strongly and positively into PC1 and this was labelled Vigilance/Attention (Table 8). Locomotion from both arena tests loaded strongly and positively and proximity to the human loaded strongly and negatively into PC2, which was labelled Bold–Shy/Activity. Lastly, response and proximity to the startle stimulus loaded strongly and positively into PC3, which was labelled Response to Startle Object.

**Figure 4:**
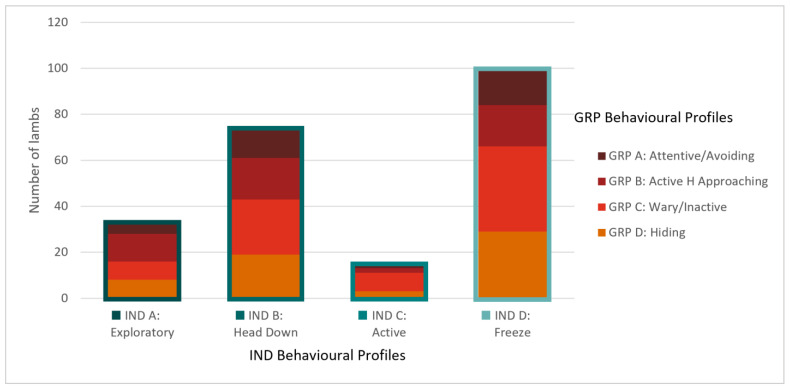
A comparison of the behavioural profiles identified from the IND (*x* axis) and GRP (stacked) testing conditions illustrates the number of lambs that fall into each profile combination. For example, of the 32 lambs classified as A: Exploratory in the IND condition, four were classified as A: Attentive/Avoiding in the GRP condition, 12 as B: Active human approaching, eight as C: Wary/Inactive, and eight as D: Hiding.

Four clusters were identified as optimal for the GRP condition (Figure 3, Table 8). Lambs in cluster A (*n* = 32) were highly vigilant and attentive in both tests, remained the furthest from the human and startle objects, exhibited the strongest startle response, and were moderately active in the startle test but not in the human test. This group was labelled the Attentive/Avoiding group. Lambs in cluster D (*n* = 59) were largely the opposite, being the least vigilant and attentive in both tests, approached the closest to the startle objects (but not the human), exhibited the lowest startle response, and had low activity (but not the least) in both tests. This group was labelled the Hiding group. Lambs in cluster B (*n* = 50) were similar to cluster A lambs in vigilance; however, they were less attentive in both tests, approached the human the closest, and were the most active in both tests. This group was labelled Active Human Approaching. The defining feature of lambs in cluster C (*n* = 77) was that they were the least active in both tests. They were also quite vigilant in both arena tests and remained away from the human. They were largely intermediate of clusters B and D for all other behaviours and were labelled the Wary/Inactive group. A comparison of the behavioural profile classifications relative to each lamb for both social conditions is illustrated in Figure 4. As an example, of the 32 lambs that were classified as A: Exploratory in the IND condition, four were classified as A: Attentive/Avoiding in the GRP condition, twelve as B: Active Human Approaching, eight as C: Wary/Inactive, and eight as D: Hiding.

## 4. Discussion

The first objective of this study was to investigate if the behavioural responses of sheep towards different stimuli were repeatable across two different socials situations when the ranking of individual animals were compared. Locomotion in the presence of a human and vocalisations, which are commonly used to identify temperament, did not show repeatability between the two social conditions. Similarities in the expression of vigilance and some attentional-orienting behaviours were found across social conditions and tests, and these behaviours should be explored further. The second objective was to explore whether the behaviour of sheep when socially isolated was indicative of the behaviour expressed when conspecifics were present. A comparison of the individual rankings between different behaviours identified some similarities; however, the implication of these relationships is unclear. A comparison of the behavioural profiles determined for each social condition also suggests no clear relationship between behaviour performed in each social condition. Sex-related differences in behavioural reactivity have been identified in sheep which indicate females are more active and spend less time near a fearful stimulus than males [16,17,18] (although see [27]). However, we found minimal effect of sex in our analyses of individual behaviours or in the distribution of males and females within the behavioural profiles.

Vocalisations and locomotion in the presence of a human are used as behavioural measures of temperament in sheep as they show strong repeatability over time. We hypothesised they would also show repeatability over social situations; however, contrary to our hypothesis, this was not the case. Vocalisations were strongly influenced by the presence of conspecifics, and our observations during testing were very similar to those described by Kilgour and Szantar-Coddington [14]: “In the group test, the silence was most striking and the ewes generally stood very close together in a group and hardly moved more than a few paces over the whole test.”. The low number of individuals to express this behaviour in either test when in the presence of conspecifics agrees with our understanding that, in the absence of a painful stimulus, sheep vocalise in response to social isolation as an attempt to reinstate social contact [17,28]. This supports our previous findings that vocalisations are indicative of an individual’s degree of sociability and are expressed in response to the absence of conspecifics [2,10]. Although these measures of temperament have consistently demonstrated high repeatability over time and, in some instances, in response to different stimuli when tested in social isolation [8,9,19,29], the lack of repeatability across social situations suggests inclusion of these behaviours in the classification of temperament should be approached with caution where that classification is applied to sheep managed in groups.

Of the other behaviours investigated for repeatability across social conditions, similarities in the rank order of expression for vigilance and attentional orienting behaviours were observed. Vigilance was not only repeatable between social conditions for both tests; this behaviour had a strong relationship between tests within each social condition, and similarities in the rank order were also found between tests and social conditions. This suggests vigilance may be indicative of a domain-general trait. A limitation of this study was that testing was not repeated at a later stage and the temporal repeatability of vigilance in either social condition could not be confirmed. However, repeatability over time [30] and contexts [31] has been found by others, which supports the suggestion that vigilance could be motivated by a domain-general trait. Attentional orienting, on the other hand, was repeatable between the social conditions in the human test but only tended towards repeatability in the startle test. While this behaviour has previously shown temporal stability towards different stimuli, this seems to only be the case where the threat remains visible [30,32]. While neither behaviour has been used previously as a measure of temperament, our findings suggest that vigilance shows promise as a repeatable trait measure across time and contexts, including different social conditions and its relationship with production outcomes, which should be explored further.

The second objective of this study was to explore the relationship between each of the IND and GRP behaviours measured to better understand if behaviour expressed in social isolation indicates how sheep will behave when conspecifics are present. Similarities in rank order between behaviours expressed under social isolation and those expressed in the presence of conspecifics suggest there may be some links between the two conditions; however, these links are not clear. For example, vocalisations across all three tests in the social isolation condition loaded together strongly in the PCA, which we have found previously [10]. This suggests that in social isolation, this behaviour is closely related regardless of the test stimuli. However, only vocalisations from the startle test showed similarities in rank order with behaviours from the group condition. Further, these similarities were with vigilance and attentional orienting in the human test only. This is despite a strong relationship between both behaviours and both tests, as identified in PC1 of the group condition. We therefore recommend interpreting these relationships with caution.

A comparison of the behavioural profiles identified under each social condition indicated no apparent link in the behaviour between the conditions, with each GRP profile consisting of a similar proportion of lambs from each IND profile. If we consider that temperament is multidimensional with a number of stable behavioural traits (for example Réale, Reader, Sol, McDougall and Dingemanse [2] discuss five, Koolhaas and Van Reenen [33] three) and that there are varying expressions of each trait, we can appreciate the complexity of all potential trait combinations. Additionally, different stimuli or circumstances may prompt stronger expressions of certain traits. For example, under social isolation, the measures recorded in the current study identified four key traits; however, in the presence of conspecifics, those same behaviours reflected three, with only one trait similar between both conditions. Therefore, the traits identified in the current study are those that are most dominantly expressed under these conditions and identifiable from the behaviours recorded, and the behavioural profiles reflect these limitations. While behavioural profiles are a useful tool to identify lambs that behave similarly and understand how different expressions of each trait interact, they may not be comparable across different situations, particularly where a new aspect/potential dimension is included. Alternatively, when considering both methods together, it may be that how a lamb behaves in social isolation is not a good indicator of how it will respond when conspecifics are present.

An interesting finding from the current study is that the temperament traits and behavioural profiles identified in social isolation closely match those identified in our previous study [10]. However, there were two key differences. The current study identified one additional temperament trait composed of the vigilance behaviours from both arena tests, which were not included in the earlier study. This trait is the defining characteristic of the behavioural profile IND B: Head Down, with lambs in this group more likely to have their head below shoulder height, as opposed to being vigilant, in both arena tests. This behaviour was observed in lambs in this group as their head was held just above the ground while the lamb remained stationary, which has been observed elsewhere in sheep where it was associated with increased plasma cortisol and lactate concentrations suggestive of a passive stress response [19,34]. As the defining trait characteristic of this profile, head down behaviour may be just as informative as vigilance, and the relationship between both extremes of this trait and the physiological and production outcomes should be explored further.

The second difference was in the expression of what we have labelled the bold–shy trait. Lambs in both the IND D: Freeze group in the current study and the equivalent group in the previous study were similar in their expression of the sociability/explore–avoid and general activity traits. However, in the current study, lambs exhibited a freeze response towards the human, as opposed to a flight response in the previous study. While fleeing and freezing can represent two different coping mechanisms or coping styles [35,36], this is unlikely to be the driver here given that these two groups have very similar behaviour in relation to the other traits. An alternative mechanism may be due to what is termed the defence cascade. Here, freezing behaviour, which may also be referred to as attentive immobility [37], precedes fleeing where responses to a threat escalate via a sequence as proximity to the threat reduces and the perception of danger increases [38,39]. It appears that the different expression of the bold–shy trait in the current study reflects the use of a stationary human, effectively switching the test from a human-avoidance to a much less aversive human-approach test, and therefore, prompting the earlier response in the defence cascade sequence. This provides further evidence that the trait we have labelled bold–shy is capturing the response to humans and supports the reliability of this testing and analysis paradigm to identify these traits under social isolation, although it should be noted that lambs for both studies were of the Merino breed from the same farm and would therefore share similar genetics.

Modelling for the GRP condition indicated that the test day order had a significant effect on several GRP behaviours, with lambs that were tested first in groups showing greater behavioural responses in the GRP condition than lambs who were tested individually first. However, it is unclear if this is due to the presentation order of the social condition or differences between the startle stimuli. As described above, to account for habituation to the tests, social condition was alternated for each group. Additionally, to avoid reduced responsiveness to the same startle stimulus, two different stimuli were used, and these were also alternated to ensure the stimulus was not tied to the condition. This has inadvertently resulted in all lambs being exposed to the umbrella first. However, as the human test was always conducted before the startle test, it is reasonable to assume the presentation order of the social condition had a greater influence on behaviours in this test than did the presentation order of the startle stimulus. It is also unclear how this issue with the study design has affected IND behaviours as modelling was only conducted on GRP behaviours. However, given that the temperament traits and behavioural profiles in the IND condition are similar to those observed previously, it appears any effect on the structure of the behavioural profiles was minimal.

A further limitation of this study is that the behavioural tests were performed concurrently and in the same order. It is therefore possible that an effect of the test order may be present and that carryover effects from the human test may have influenced behaviour in the latter tests.

## 5. Conclusions

The findings from the current study indicate that conclusions drawn from testing sheep individually may not be applicable for sheep that are managed in groups. Social isolation is useful for generating the degree of behavioural variability needed to differentiate distinct temperament classifications and commonly used behavioural indicators of temperament in sheep show high repeatability under this condition. However, we were not able to confirm the repeatability of vocalisations or locomotory behaviours between social situations, with vocalisations rarely performed when conspecifics were present. Further, we have demonstrated that behaviour expressed under social isolation is not related to behaviour expressed in the presence of conspecifics. We found no relationship between individual behaviours or behavioural profiles across the two social situations. The experience of being socially isolated is known to be highly stressful in sheep and our results suggest this has a significant effect on the behavioural variability seen. Temperament classifications applied to socially isolated sheep may be meaningless when applied to settings where sheep are managed in groups; however, it may still be useful for situations where sheep need to be isolated or are housed individually. Vigilance shows promise as a measure of an underlying trait that is stable across social contexts and should be explored further to understand how it relates to welfare outcomes and desirable production traits.

## Figures and Tables

**Table 1:** All behaviours for the human (H) and startle (S) tests were recorded for both the individual (IND) and group (GRP) conditions. Behaviours for the Isolation Box (IB) test were recorded for the IND condition only. No interactions with any stimulus occurred under the GRP condition.

Behaviour	Description	H Test	S Test	IB Test
Vocalisations	Number of vocalisations (low- and high-pitched bleats).	IND GRP	IND GRP	IND
Escape attempts	Number of jumps against the arena or isolation box walls (two feet or less remain on the ground).	IND GRP	IND GRP	IND
Locomotion	Number of single front-foot steps.	IND GRP	IND GRP	IND
Vigilance	Time spent with head at or above shoulder height.	IND GRP	IND GRP	
Attention to stimulus	Time spent with head oriented towards the stimulus (human/umbrella/ball).	IND GRP	IND GRP	
Proximity to stimulus	Closest zone to stimulus the lamb entered: 1 = came within 0–1 m 2 = came within 1–2 m 3 = came within 2–3 m 4 = stayed 3 m + away	IND GRP	IND GRP	
Interactions with the stimulus	If contact was made with the stimulus (human/umbrella/ball) using any body part (yes/no).	IND	IND	
Startle response	Magnitude of startle response: 0 = did not startle 1 = jumped/startled but took no steps 2 = took steps but moved <1 square 3 = ran or moved >1 square 4 = fled and may have attempted escape		IND GRP	
Turns	Number of times lamb turned body 180 degrees to face the opposite door of the isolation box			IND

**Table 2:** Descriptive statistics for behaviours measured under both social conditions. For proximity to the stimulus measures, a higher number indicates a greater distance from the stimulus (human/umbrella/ball). Similarly, for the startle response, a higher number indicates greater behavioural reactivity in response to being startled.

Test	Behaviour	Unit	Number of Lambs to Perform Behaviour		Mean per Individual		Range (min, max)	
			IND	GRP	IND	GRP	IND	GRP
Human	Vocalisations	count	179 (81%)	7 (3%)	18.5	0.2	(0, 75)	(0, 13)
	Escape Attempts	count	112 (51%)	4 (2%)	1.4	0.02	(0, 19)	(0, 1)
	Locomotion	count	218 (99%)	215 (98%)	117	34.6	(0, 381)	(1, 101)
	Vigilance	seconds	220 (100%)	220 (100%)	223	170	(150.8, 240)	(1.1, 240)
	Attention to Human	seconds	220 (100%)	215 (98%)	86.4	31.9	(18.2, 181.5)	(0, 156.2)
	Proximity to Human	1–4	220 (100%)	220 (100%)	2.3	3.6	(1, 4)	(2, 4)
Startle	Vocalisations	count	170 (77%)	8 (4%)	18.7	0.2	(0, 87)	(0, 16)
	Escape Attempts	count	74 (34%)	7 (3%)	0.8	0.03	(0, 9)	(0, 1)
	Locomotion	count	220 (100%)	217 (99%)	112	32.3	(4, 359)	(0, 94)
	Vigilance	seconds	220 (100%)	220 (100%)	216	176	(124.8, 240)	(6.3, 240)
	Attention to Object	seconds	220 (100%)	209 (95%)	63.4	32.8	(7.8, 175.2)	(0, 180.1)
	Proximity to Object	1–4	220 (100%)	220 (100%)	2.1	3.3	(1, 4)	(1, 4)
	Startle Response	0–4	211 (96%)	157 (71%)	2.4	1.6	(0, 4)	(0, 4)

**Table 3:** Repeatability estimates for behavioural measures using ranked data to determine stability of expression between two different social conditions: when tested individually (IND as baseline covariate) and in the presence of conspecifics (GRP as outcome variable). The effect of test day order (alternating social conditions) is also presented where applicable.

Test	Behaviour	IND Condition				Test Day Order			
		Estimate	*p*-Value	2.5% CI	97.5% CI	Estimate	*p*-Value	2.5% CI	97.5% CI
Human	Locomotion *	0.05	0.288	−0.05	0.15	-	-	-	-
	Vigilance	0.31	<0.001	0.2	0.42	44.6	<0.001	24.49	64.64
	Attention to Human	0.2	<0.001	0.09	0.31	52.7	<0.001	31.34	74.13
	Proximity to Human	0.05	0.152	−0.02	0.12	-	-	-	-
Startle	Locomotion	0.1	0.048	0.002	0.2	-	-	-	-
	Vigilance	0.13	0.018	0.03	0.24	57.2	<0.001	36.71	77.63
	Attention to Object	0.12	0.065	−0.01	0.24	46.4	<0.001	25.61	67.1
	Proximity to Object	0.01	0.639	−0.03	0.05	1.4	<0.001	1.02	1.69
	Startle Response	0.05	0.736	−0.23	0.33	4.2	<0.001	2.3	6.08

* Behaviour has shown temporal stability in sheep when tested in social isolation.

**Table 4:** Estimates of IND behaviours that showed a significant loading on GRP behaviours in the human (H) and startle (S) tests. The effects of test day order (alternating social conditions), sex, and weight at weaning are presented where applicable.

GRP Behaviour	IND Behaviour/Fixed Effect	Estimate	*p*-Value	2.5% CI	97.5% CI
Vigilance (H)	Vocalisations (S) *	−0.16	0.01	−0.27	−0.05
	Vigilance (S)	0.14	0.03	0.02	0.26
	Test Day Order	48.19	<0.001	28.36	68.05
Attention to human	Vocalisations (S) *	−0.12	0.04	−0.23	−0.01
	Escape Attempts (H) *	0.16	0.01	0.04	0.28
	Test Day Order	59.61	<0.001	38.51	80.66
Proximity to human	Interactions (H)	−0.002	0.03	−0.003	−0.0001
	IND S Steps	−0.001	0.01	−0.002	−0.0003
Vigilance (S)	Vigilance (H)	0.2	<0.001	0.09	0.3
	Test Day Order	55.81	<0.001	35.35	76.25
Attention to S object	Locomotion (IB) *	0.16	0.01	0.04	0.29
	Test Day Order	39.98	<0.001	20.9	59.07
	Sex	−19.77	0.03	−36.73	−2.79
	Weight at weaning	2.29	0.02	0.33	4.3
Proximity to S object	Test Day Order	1.35	<0.001	1.02	1.69
Locomotion	Proximity to S Object	8.11	0.04	0.59	15.63
S response	Locomotion (H) *	−0.01	0.04	−0.01	−0.0004
	Test Day Order	4.14	<0.001	2.57	5.71

* Behaviour has shown temporal stability in sheep when tested in social isolation.

**Table 5:** The magnitude and direction of the behavioural loadings within the four retained principal components (PCs) for the IND social condition. Behaviours with an absolute loading >0.45 (bolded) have been considered when interpreting that PC.

IND Behaviours	PC1 Sociability/Explore–Avoid	PC2 General Activity	PC3 Bold–Shy	PC4 Vigilance	h^2^
Vocalisations (S)	**0.85**	−0.09	0.07	0.04	0.75
Vocalisations (H)	**0.84**	−0.18	0.11	0.07	0.75
Proximity to Human	**−0.74**	−0.12	−0.08	0.18	0.6
Interactions (H)	**0.71**	0.05	0.04	0.04	0.5
Interactions (S)	**0.61**	0.41	−0.03	−0.22	0.58
Proximity to S Object	**−0.6**	−0.35	−0.07	0.38	0.63
Vocalisations (IB)	**0.6**	−0.09	−0.02	0.29	0.45
Locomotion (S)	**0.49**	0.42	0.38	−0.22	0.6
Locomotion (IB)	0.09	**0.79**	0.13	0.1	0.66
Turns (IB)	−0.03	**0.79**	0.05	0.04	0.63
Escape Attempts (IB)	−0.17	0.36	0.11	0.32	0.27
Escape Attempts (S)	−0.02	0.15	**0.76**	0.17	0.63
Escape Attempts (H)	−0.2	0.2	**0.68**	0.3	0.63
Locomotion (H)	0.38	0.39	**0.58**	−0.04	0.64
Attention to Human	−0.21	0.08	**−0.58**	0.35	0.5
Attention to S Object	−0.14	0.06	−0.43	0.32	0.31
Vigilance (S)	0.07	0	0	**0.77**	0.6
Vigilance (H)	0.06	0.09	0.01	**0.74**	0.55
Proportion Variance	0.23	0.12	0.12	0.11	
Cumulative Variance	0.23	0.35	0.46	0.57	

**Table 6:** Cluster means for the IND condition illustrate differences in behavioural expressions within each behavioural profile. Behaviours are listed according to how they loaded into the four principal components for the IND condition and defining/differentiating behaviours are bolded.

	IND Behaviour	Cluster IND A: Exploratory	Cluster IND B: Head Down	Cluster IND C: Active	Cluster IND D: Freeze
PC1	Vocalisations (S)	**1.33**	0.16	−0.11	**−0.53**
	Vocalisations (H)	**1.36**	0.13	−0.30	**−0.49**
	Proximity to Human	**−1.38**	−0.28	0.14	**0.63**
	Interactions (H)	**2.23**	−0.41	−0.21	−0.39
	Interactions (S)	**1.00**	0.47	0.09	**−0.68**
	Proximity to S Object	−0.72	−0.67	−0.24	**0.76**
	Vocalisations (IB)	**1.06**	−0.15	−0.15	−0.21
	Locomotion (S)	0.67	0.54	**0.91**	**−0.74**
PC2	Locomotion (IB)	0.37	0.18	**0.92**	**−0.38**
	Turns (IB)	0.02	0.18	**0.76**	−0.25
	Escape Attempts (IB)	0.05	−0.21	**0.68**	0.04
PC3	Escape Attempts (S)	−0.02	−0.17	**2.88**	−0.28
	Escape Attempts (H)	−0.20	−0.25	**2.54**	−0.11
	Locomotion (H)	0.67	0.26	**1.37**	**−0.60**
	Attention to Human	−0.36	−0.28	−0.34	**0.37**
	Attention to S Object	−0.22	−0.17	**−0.41**	**0.26**
PC4	Vigilance (S)	0.10	**−0.30**	**0.50**	0.12
	Vigilance (H)	0.01	**−0.24**	**0.53**	0.10
Males		16	30	8	52
Females		16	43	6	47
Total		32	73	14	99

**Table 7:** The magnitude and direction of the behavioural loadings within the three retained principal components (PCs) for the GRP social condition. Behaviours with an absolute loading >0.45 (bolded) have been considered when interpreting that PC.

GRP Behaviours	PC1 Attention/ Vigilance	PC2 Bold–Shy/ Activity	PC3 Response to S Object	h^2^
Vigilance (H)	**0.77**	0.2	0.19	0.67
Attention to Human	**0.75**	−0.11	0.06	0.58
Vigilance (S)	**0.73**	0.18	0.28	0.64
Attention to S Object	**0.66**	−0.02	0.05	0.44
Locomotion (H)	0.04	**0.84**	0.03	0.71
Proximity to Human	0.11	**−0.75**	0.18	0.61
Locomotion (S)	0.18	**0.6**	0.11	0.41
Startle Response (S)	0.09	0.01	**0.85**	0.72
Proximity to S Object	0.22	−0.03	**0.71**	0.55
Proportion Variance	0.25	0.19	0.15	
Cumulative Variance	0.25	0.44	0.96	

**Table 8:** Cluster means for the GRP condition illustrate differences in behavioural expressions within each behavioural profile. Behaviours are listed according to how they loaded into the three principal components for the GRP condition and defining/differentiating behaviours are bolded.

	GRP Behaviour	Cluster GRP A: Attentive/Avoiding	Cluster GRP B: Active H approaching	Cluster GRP C: Wary/Inactive	Cluster GRP D: Hiding
PC1	Vigilance (H)	0.68	0.49	0.37	**−1.27**
	Attention to Human	**1.58**	−0.03	−0.16	**−0.62**
	Vigilance (S)	0.57	0.56	0.43	**−1.35**
	Attention to S Object	**1.35**	0.17	−0.24	**−0.56**
PC2	Locomotion (H)	−0.21	**1.09**	**−0.46**	−0.21
	Proximity to Human	**0.61**	**−1.06**	0.42	0.02
	Locomotion (S)	**0.52**	**0.61**	**−0.38**	−0.31
PC3	Startle Response (S)	**0.61**	0.01	0.12	**−0.50**
	Proximity to S Object	**0.61**	0.24	0.05	**−0.60**
Males		18	31	37	26
Females		14	19	40	33
Total		32	50	77	59

## Data Availability

The data presented in this study are available on request from the corresponding author.
